# Dynamical manipulation of electromagnetic polarization using anisotropic meta-mirror

**DOI:** 10.1038/srep30771

**Published:** 2016-07-29

**Authors:** Jianhua Cui, Cheng Huang, Wenbo Pan, Mingbo Pu, Yinghui Guo, Xiangang Luo

**Affiliations:** 1 State Key Laboratory of Optical Technologies on Nano-Fabrication and Micro-Engineering, Institute of Optics and Electronics, Chinese Academy of Science, P. O. Box 350, Chengdu 610209, China

## Abstract

Polarization control of electromagnetic wave is very important in many fields. Here, we propose an active meta-mirror to dynamically manipulate electromagnetic polarization state at a broad band. This meta-mirror is composed of a double-layered metallic pattern backed by a metallic flat plate, and the active elements of PIN diodes are integrated into the meta-atom to control the reflection phase difference between two orthogonal polarization modes. Through switching the operating state of the PIN diodes, the meta-mirror is expected to achieve three polarization states which are left-handed, right-handed circular polarizations and linear polarization, respectively. We fabricated this active meta-mirror and validated its polarization conversion performance by measurement. The linearly polarized incident wave can be dynamically converted to right-handed or left-handed circular polarization in the frequency range between 3.4 and 8.8 GHz with the average loss of 1 dB. Furthermore, it also can keep its initial linear polarization state.

Polarization state is of great importance in many electromagnetic (EM) devices since a majority of EM phenomenon is polarization sensitive. A wave plate, based on a birefringent crystal with specific orientation and thickness, is a traditional method to manipulate polarization. It can achieve linear to circular polarization with different handedness by the superposition of two orthogonal linearly polarized waves with a certain phase shift due to difference of refractive index along the two axes. The handedness is mainly dependent on the phase difference that is associated with the crystal thickness. As the difference between refractive indexes is typically very small, a large thickness is often required. In addition, the polarization conversion is only restricted to a narrow bandwidth because the produced phase shift between two orthogonal polarization modes is frequency dependent. It is still worth noting that the traditional wave plate cannot dynamically manipulate EM polarization states.

With the great capacity to manipulate the EM wave, metamaterials or meta-surfaces have caused much interest and resulted in many intriguing applications, such as negative refraction^1^,^2^, flat lens^3^,^4^, Fano resonance^5^, and invisibility cloak^6^,^7^. For the polarization control, both chiral metamaterials^8^,^9^,^10^,^11^,^12^,^13^,^14^ and anisotropic metamaterials^15^,^16^,^17^,^18^ behave strong capacities. Due to the strong coupling between electric and magnetic fields, chiral metamaterials exhibit two properties, such as circular dichroism^8^,^9^,^10^,^11^ and optical rotation^12^,^13^,^14^. It can not only transform a linearly-polarized (LP) wave into a circular polarized (CP) wave with different handedness at different frequencies, but also rotate the incident wave by a certain angle. However, the most of chiral metamaterials only operate in a narrow bandwidth because of highly resonant nature of meta-atoms. Although several methods, including multilayer^19^,^20^ and helix structures^21^,^22^, have been reported to extend the operation bandwidth, the high loss of the chiral metamaterial is still dissatisfactory, especially for the CP chiral metamaterial. The anisotropic metamaterial adopts the similar working principle of the briefrigent crystal, which can independently tune the transmission or reflection phases (*φ*_*1*_ and *φ*_*2*_) along two orthogonal axes. By designing the phase difference *Δφ* = *φ*_*1*_ − *φ*_*2*_, the anisotropic metamaterials under illumination of a linearly-polarized wave can realize the different outgoing polarization states, including left-handed circular polarization (LHCP) at case of *Δφ* = π/2, right-handed circular polarization (RHCP) at case of *Δφ* = 3π/2 and linear polarization (LP) at case of *Δφ* = π, assuming no material loss is generated. However, the same reason of the naturally resonance in the cell causes the polarization conversion of this kind of metamaterial limited to a small bandwidth. In order to address this issue, a nascent strategy of dispersion management was proposed and applied to a single dimension of a reflective meta-surface, and thus, a LP wave was achromatically converted to its cross-polarization state over a 3:1 fractional bandwidth with transformation efficiency of 90%^17^. More recently, the bandwidth of polarization conversion was further extended to 5:1 octaves by implementing the dispersion management in the two dimensions of the meta-surface for achieving the ideal phase retardation on two orthogonal directions^18^. Despite of the great progress, the above broadband polarization transformer only can achieve the single outgoing polarization state. Therefore, dynamical metamaterial has been developed to satisfy the multi-polarization requirement. The active elements or tunable materials, such as microelectromechanical systems^23^,^24^ (MEMS), PIN diodes^25^, photoactive medium^26^ and grapheme^27^, have been utilized in the design of meta-atom. With outside stimuli, the metamaterial is expected to achieve real-time manipulation of polarization states. However, the loss and narrow bandwidth for the dynamical polarization transformation severely impede their further development. So it is still a great challenge to actively manipulate polarization states with low loss in a broad band.

In this article, an actively controlled meta-mirror is proposed to manipulate the polarization states of the reflected wave in a broad band. It can convert linearly-polarized wave to LHCP, RHCP or originally LP wave by tuning the bias voltage applied to the PIN diodes in the meta-atoms. The dispersion management is employed in the two dimensions of the proposed meta-mirror to achieve ideal phase retardation for achromatic polarization conversion. Through numerical simulation and experimental measurement, we demonstrate the strong ability of the designed meta-mirror in dynamical polarization manipulation over a wide band.

## Results

The proposed meta-mirror is generally composed of an anisotropic metallic pattern and a metallic flat plate with a dielectric spacer between them. By specially designing an anisotropic metallic pattern, any desired reflection phase difference between *x*- and *y*-directions can be produced. Since the polarization transformation is mainly dependent on this phase difference, the geometrical design of the anisotropic cell plays a key role in the polarization characteristic of the meta-mirror. We can use transfer matrix method to calculate the reflection phase of the anisotropic meta-mirror along *x*- and *y*-directions, respectively.


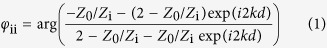


**Figure 1:**
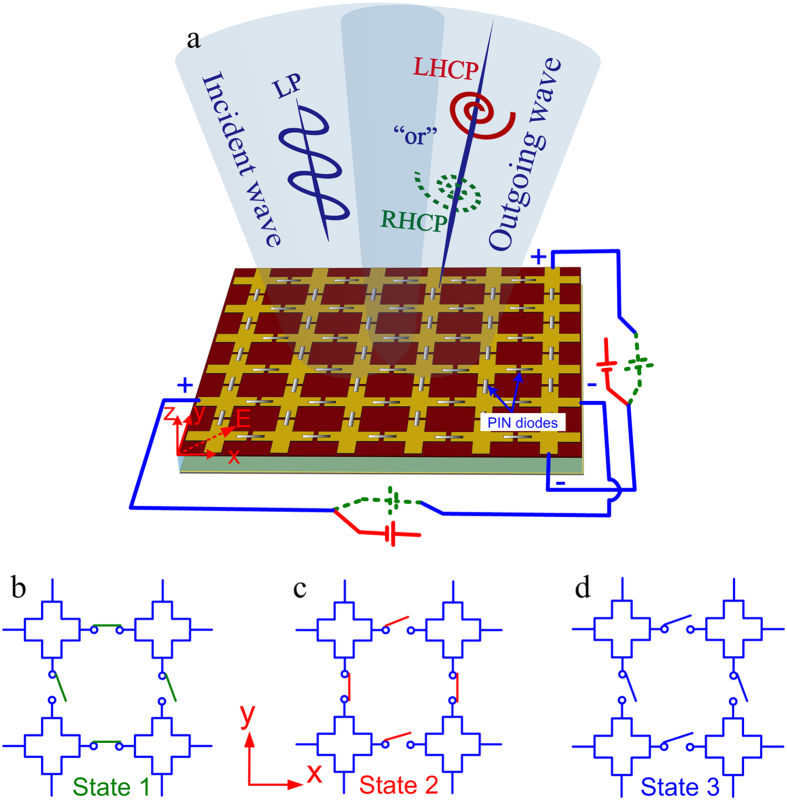
Operating principle diagram of the active meta-mirror. (**a**) Schematic model of the active meta-mirror. (**b**–**d**) Equivalent structures of the meta-mirror at three operating states.

Where *k* is the wave vector in free space and *d* is the thickness of dielectric spacer. *i* = *x*, *y* represent the electric field polarized along the *x*- and *y*-direction, respectively. *Z*_*i*_(*ω*) indicates the surface impedance of the meta-mirror, and *Z*_*0*_ = 377 Ω is the impedance of free space. Both *φ*_*xx*_ and *φ*_*yy*_ are frequency dependent, and the transformation of LP wave to LHCP or RHCP wave would be produced assuming that *Δφ*(*ω*) = *φ*_*xx*_(*ω*) − *φ*_*yy*_(*ω*) = 90° or −90° and no material loss is generated. In order to construct the active meta-mirror, the PIN diode is integrated into the design of the meta-atoms. Figure 1(a) shows the general operating principle of the active meta-mirror. It is composed of a single-layer periodic cross metallic strip structure backed by a metallic flat plate. The active elements of PIN diodes are loaded on the gaps of the cross metallic strips along both *x*- and *y*- directions where they are independently controlled by the bias voltage, so that we can dynamically tune the phase difference between these two orthogonal directions. When the proposed active meta-mirror is illuminated by an LP wave with electric field polarizing at 45 degree with respect to the *x*-axis, three different polarization states of the outgoing wave could be obtained. As Fig. 1(b) shows, if the PIN diodes are switched on along the *x*-direction and turned off along the *y*- direction at the state 1, the phase difference of −90° (*Δφ*(*ω*) = *φ*_*xx*_(*ω*) *−* *φ*_*yy*_(*ω*)) could be constructed by optimizing the metallic pattern, and then the meta-mirror would convert the LP incident wave into RHCP reflected wave. When all the PIN diodes are changed into their opposite states (state2), as shown in Fig. 1(c), the phase difference of 90° could be obtained, resulting in the production of the LHCP reflected wave. If all the PIN diodes are switched off at the state 3, as seen in Fig. 1(d), the meta-mirror would become isotropic, and original LP state is expected to be reserved since no phase shift is produced on the two orthogonal directions.

**Figure 2:**
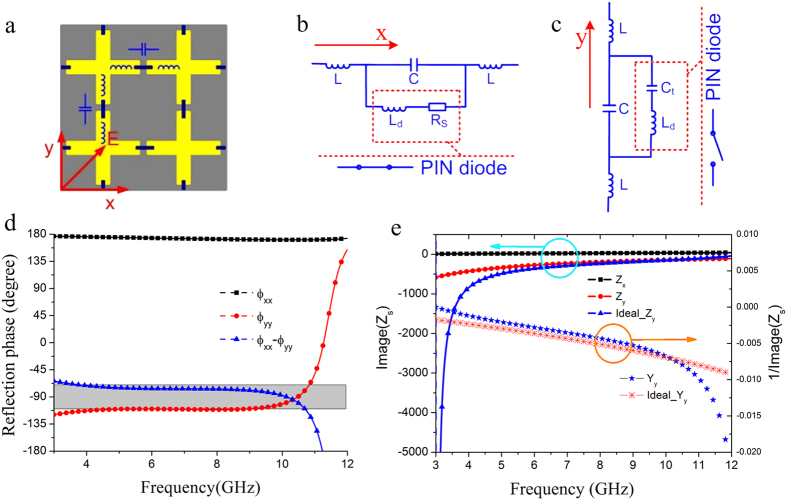
Simple model of the active meta-mirror and its simulation results. (**a**) Schematic of the simple model for the active meta-surface. (**b**,**c**) Equivalent circuits of this simple model at *x*- and *y*- directions, respectively. (**d**) Reflection phase distribution *φ*_*xx*_, *φ*_*yy*_ and *φ*_*xx*_ − *φ*_*yy*_ with the fitting parameter results of *L* = 0.1 nH, *C* = 5 fF. (**e**) Effective impedance *Z*_*x*_ and *Z*_*y*_ calculated from formula (2–3), and the calculated ideal *Z*_*y*_ for the given *Z*_*x*_ (*Δφ*(*ω*) = *−90°*) using formula (1). The admittance curves of *Y*_*y*_ and the ideal *Y*_*y*_ are also given for observation.

To verify the feasibility of the active meta-mirror at a broad band, the above simple design is adopted for the theoretical analysis. M/A-COM Flip Chip MA4SPS502 is selected for the loaded PIN diodes. Its total capacitance is *C*_*t*_ = 0.09 pF @ −40 V for reverse bias, the inductance of this diode is *L*_*d*_ = 0.35 nH, while the series resistance is *Rs* = 2.4 Ω for a forward bias current of 20 mA. The working central frequency of this meta-mirror is designed at 6 GHz, and an air spacer is inserted between the metallic pattern layer and metallic plate. In order to achieve wideband polarization conversion, the air spacer thickness cannot be too small, or else the strong magnetic coupling between the meta-surface and ground plane would result in non-constant phase gradient, causing the limited bandwidth. Here, the thickness is designed to be 12 m that is about quarter of wavelength at the central frequency. There is almost no coupling between the metallic pattern and metallic flat plate. In addition, assuming that the PIN diodes are switched on along the *x*-direction and they are in the OFF state along the *y*-direction, the equivalent circuit of this meta-mirror along these two directions can be obtained, as shown in Fig. 2(b,c), respectively. The inductors (*L*) and capacitors (*C*) are due to the cross metallic strip and its gap, respectively. Therefore, the frequency-dependent impedance for *Z*_*x*_ and *Z*_*y*_ can be expressed as follows:


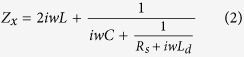



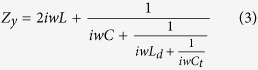


In the microwave domain, 3-dB axial ratio is generally adopted to express the bandwidth of circularly polarized (CP) wave. Assuming that no material loss is generated, that is, the meta-mirror can reflect all the incoming wave energy at both *x*- and *y*- polarizations, the reflection phase difference of *Δφ*(*ω*) between these two polarizations can be calculated to be located in range of (−90° − 36.75°, −90° + 36.75°) or (90° − 36.75°, 90° + 36.75°)^28^. Considering fabrication tolerance and measurement error, the phase difference varying range is reduced to (±90° − 20°, ±90° + 20°) to define the bandwidth of the CP wave in simulation. Here, we take LP-RHCP conversion as an example to investigate this simple model of the active meta-mirror. According to the formula (1–3), the phase difference *Δφ*(*ω*) mainly depends on the values of *L* and *C* which can be evaluated by fitting *Δφ*(*ω*) with the ideal phase difference of −90°. When the circuit parameters satisfy (*L*, *C*) = (0.1 nH, 5 fF), the phase difference *Δφ*(*ω*) fluctuates in the range of (90° − 20°, 90° + 20°) at a wide band from 3.3 GHz to 11.3 GHz, as seen in Fig. 2(d). Additionally, the corresponding impedance *Z*_*x*_, *Z*_*y*_ can be calculated by formula (2–3), and the ideal impedance *Z*_*y*_ for the given *Z*_*x*_ could be derived from formula (1) as well. As Fig. 2(e) shows, the calculated *Z*_*y*_ almost approaches the ideal *Z*_*y*_ at a wide frequency range. Therefore, the above calculation results of the simple model for the active meta-mirror have fully demonstrated its capability of dynamical polarization conversion at a wide band.

**Figure 3:**
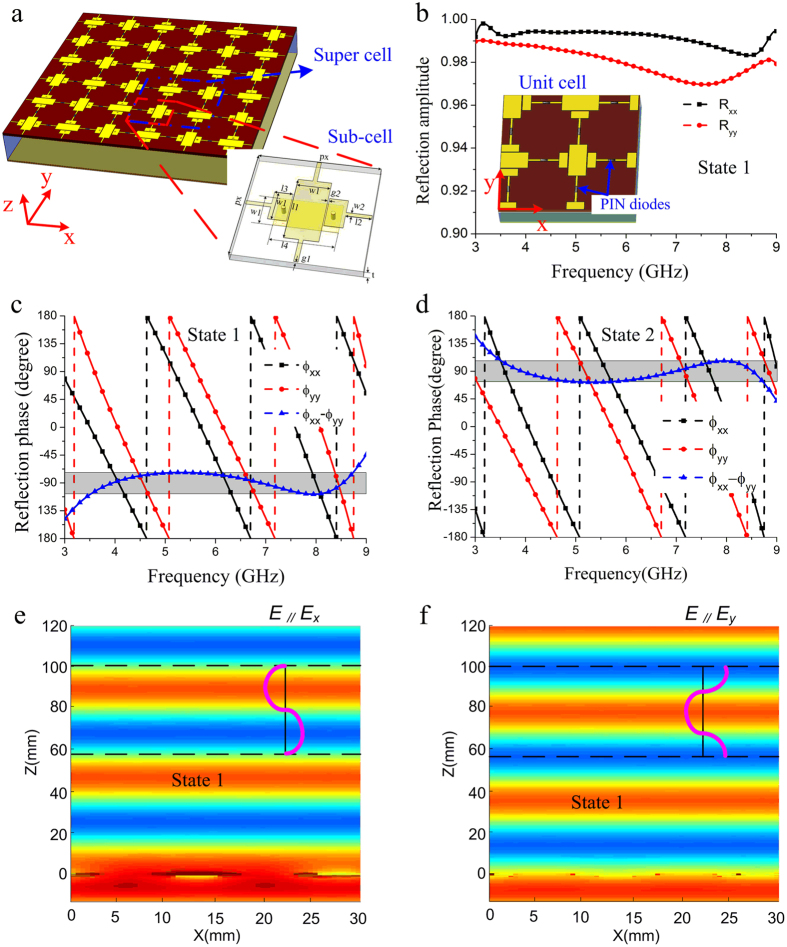
The designed active meta-mirror and its simulation results. (**a**) 3D-view of the designed active meta-mirror. Its sub-cell and super-cell are marked in different line boxes. (**b**) Simulated reflection amplitude of *x*- and *y*-polarizations at the state 1. The unit cell in simulation is given in the inset of this picture. (**c**) Simulated reflection phase of *x*- and *y*-polarizations at the state 1. The phase difference range of (−90° − 20°, −90° + 20°) is indicated by a gray shaded region. (**d**) Simulated reflection phase of *x*- and *y*-polarizations at the state 2. The phase difference range of (90° − 20°, 90° + 20°) is indicated by a gray shaded region. (**e**,**f**) Simulated electric-field distributions of *x*- and *y*- polarized reflection waves at the state 1, respectively.

Figure 3 shows the geometry of the designed wideband meta-mirror that can dynamically manipulate polarization states of reflection wave. The super cell of this meta-mirror is composed of four sub-cells which are arranged to possess C4 symmetry. The sub-cell structure consists of a double-layered metallic pattern printed on both sides of a dielectric substrate, as shown in the inset of Fig. 3(a). There is a continuous metallic strip along the *y*-direction. In order to avoid the crossing of two metallic strips between *x*- and *y*- directions, the metallic strip along the *x*-direction is constructed by three rectangular patches through two metalized via-holes. The PIN diodes of M/A-COM Flip Chip MA4SPS502 are inserted on the gap between all the adjacent sub-cells. For the LP-CP transformation, the operating state of the PIN diodes at the *x*-direction would be different from that at the *y*-direction. If the incident LP state needs to be reserved, all the PIN diodes should work in the same states. The dielectric substrate selected to support the metallic structure is 1 mm thick F4B with relative permittivity *ε*_*r*_ of 2.65 and tangent loss of 0.001. The period of the unit cell is set to be *px* = 15 mm. In addition, there is an air spacer with a thickness of 12 mm between the dielectric substrate and metallic flat plate. In order to verify the reflection characteristics of this meta-mirror, numerical simulation is carried out by using a commercial software CST microwave studio 2014. The unit cell for simulation is given in inset of Fig. 3(b). Periodic boundary condition is set to its *x* and *y* sides, and we adopt *x*- and *y*- polarized wave, respectively, as the exciting source to obtain its reflection characteristic. The geometric parameters of the double-layered metallic patterns are optimized as follows: *l1* = 8.2 mm*, l2* = 1.95 mm*, l3* = 3.25 mm*, l4* = 7.6 mm*, w1* = 4 mm, *w2* = 0.5 mm*, g* = 0.3 mm and *g2* = 0.15 mm. Figure 3(b) shows the reflection coefficient of this meta-mirror at the state 1. It is seen that reflection amplitudes for both *x*- and *y*- polarizations are larger than 0.95, which means that the incoming wave is almost totally reflected by this meta-mirror at these two polarizations. However, it is seen in Fig. 3(c) that their reflection phases is obviously different, and the phase difference between them fluctuates in the range of (−90° − 20°, −90° + 20°) from 3.6 GHz to 8.7 GHz. When the meta-mirror is tuned to operate at the state 2, the similar results are expected to be obtained, and the phase difference between *x*- and *y*-polarizations would be located in the range of (90° − 20°, 90° + 20°) at the same frequency band, as seen in Fig. 3(d). Figure 3(e,f) show the simulated electric field distribution of the *x*-polarized and *y*-polarized reflection waves at the state 1, respectively. It can be seen that both *x*- and *y*-polarized incident waves are vertically reflected and their wave fronts have obvious phase difference. The reflection phase of the *y*-polarized waves is 90° ahead compared to the *x*-polarized reflection waves. Hence, the RHCP reflection wave would be produced when the meta-mirror is illuminated by a normal incident wave with electric field along the structure diagonal. Due to rotational symmetry for the geometrical structure of the designed meta-mirror, the opposite handedness of the CP wave could be realized at the state 2 where the reflection wave of the *x* component is designed to advance the *y* component by 90°.

**Figure 4:**
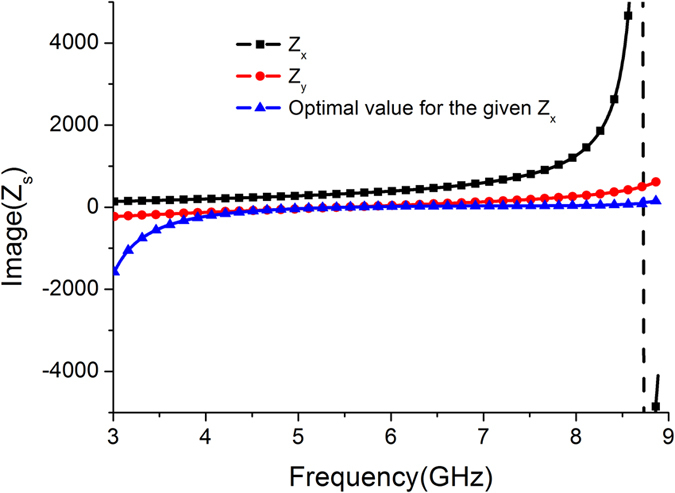
Effective retrieved impedance *Z*_*x*_ (black dash line) and *Z*_*y*_ (red dash line). Blue dash line shows the optimal impedance *Z*_*y*_ for the given *Z*_*x*_. The real part of impedance is zero since no material loss is supposed.

**Figure 5:**
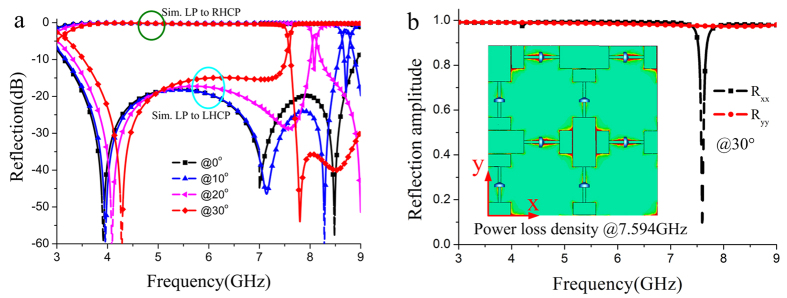
Simulation results of the active meta-mirror under oblique illumination. (**a**) Simulated circularly polarized reflection spectra of the active meta-mirror under illumination with different oblique incident angles at the state 1. (**b**) Simulated *x*- and *y*- polarized reflection amplitudes at the oblique incident angle of 30°. The inset of this picture depicts the *x*-polarized power loss density at 7.594 GHz.

According to the simulation results given in Fig. 3(b,c), we can further calculate the effective sheet impedance through transfer matrix method analysis that is well described in ref. 18. Figure 4 depicts the retrieved results for the effective anisotropic impedances *Z*_*x*_ and *Z*_*y*_ at the state 1. The optimal impedance *Z*_*y*_ that is calculated for the given *Z*_*x*_ to construct a CP wave is also given as comparison. It is seen that the retrieved impedance *Z*_*y*_ of this meta-mirror agrees well with the optimal impedance value over a wide frequency range of interest. For further investigation of the applicability of the meta-mirror under oblique incidence, the reflection characteristics for different oblique incident angles of 10°, 20° and 30° at the state 1 are studied, as seen in Fig. 5(a). With the increase of oblique incident angle, there is a strong resonance peak that is gradually shifted towards lower frequency. The polarization conversion effect around this resonance frequency is deteriorated, but the designed meta-mirror still possesses the capacity of the wideband polarization conversion. The relative bandwidth for CP outgoing wave is beyond 60% for all the oblique incident angles. In order to understand the production of the above resonance peak, we investigate the *x*- and *y*-polarized reflection characteristic of this meta-mirror with oblique incident angle of 30°, and the corresponding result is given in Fig. 5(b). As it shows, a strong absorption phenomenon is produced in the *x*- polarization at the frequency of 7.594 GHz, which means that there is almost no reflection wave energy. The inset of Fig. 5 (b) shows the power loss density distribution for the *x*- polarization at the absorbing frequency. It is obvious that the high power loss density is located along *x-*direction, especially at the gaps where the PIN diodes are loaded. So we consider that most of incident wave is dissipated on the series resistor of the PIN diodes and then converted into heat energy.

**Figure 6:**
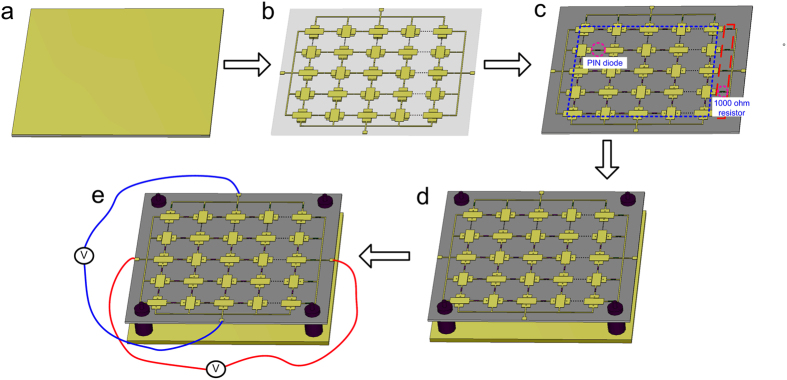
Schematic fabrication process flow. (**a**) Starting with a 1 mm thick double-face copper clad laminate. (**b**) Etching the metallic pattern through printed circuit board technique. (**c**) Soldering the PIN diodes and 1000 ohm resistors on this sample. (**d**) Integrating the sample with a metallic flat plate. (**e**) Controlling the meta-mirror with a two-way DC voltage source.

**Figure 7:**
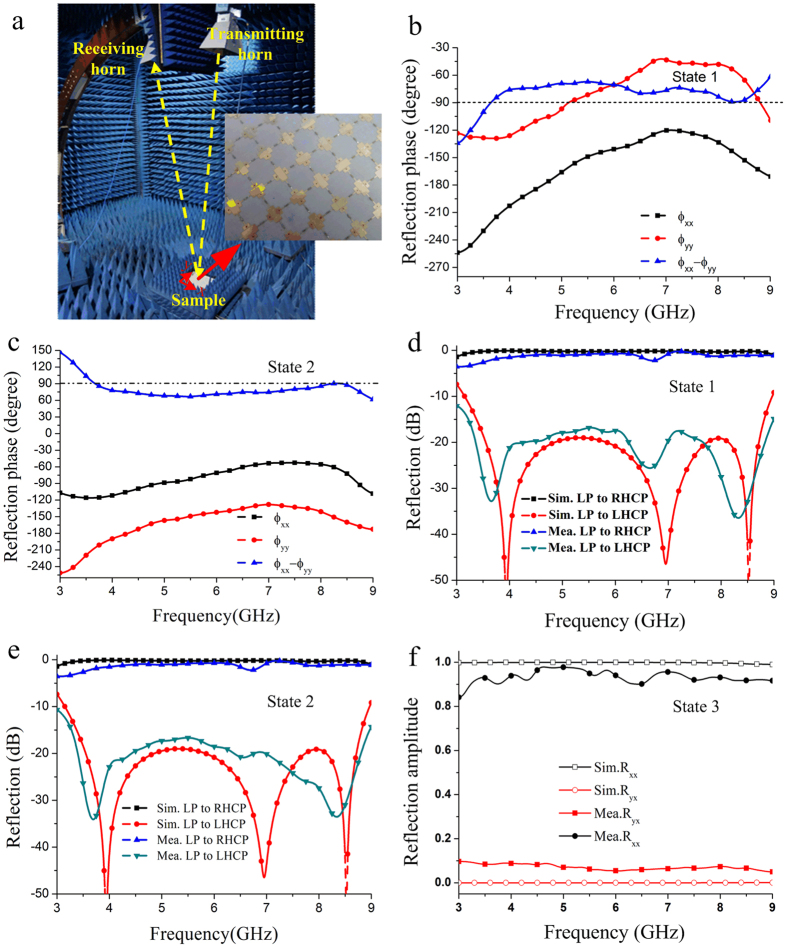
Experimental verification of the active meta-mirror. (**a**) Photograph of the measurement setup and fabricated sample. (**b**,**c**) Measured reflection phase distributions at the state 1 and state 2, respectively. (**d**,**e**) Measured and simulated CP reflection spectra at the state 1 and state 2, respectively. (**f**) Measured and simulated linearly polarized reflection spectra at the state 3.

In order to validate the simulation results of the designed meta-mirror, the sample with a dimension of 360 mm × 360 mm was fabricated and its schematic fabrication process flow is depicted in Fig. 6. Firstly, a 1 mm thick double-face copper clad laminate with relative permittivity *ε*_*r*_ of 2.65 is selected and the metallic pattern of the meta-mirror is etched on its two sides by using printed circuit board (PCB) technology. Then, the PIN diodes are soldered between the adjacent sub-cells of the meta-mirror, and 1000 ohm resistors are used between each branch of the metallic structure and the direct current (DC) feeding line for producing the same amount of current for all the diodes and protecting the diodes as well. The fabricated sample is placed at a height of 12 mm away from a metallic flat plate, and four nylon spacers are utilized to support the whole meta-mirror. Finally, a two-way DC voltage source is adopted to control the states of PIN diodes. The polarization conversion characteristic of the meta-mirror was measured in the anechoic chamber. Figure 7(a) shows the reflection measurement setup. Two wideband horn antennas connected to the two ports of a vector network analyzer R&S ZVA40 are selected as a transmitter and receiver, respectively. Their incidence and reflection angles are fixed as 5° to make a good approximation of the normal incidence. The square sample is located in the central stage of the whole measurement setup, and its diagonal is parallel to the *x*-axis. For the LP-CP conversion states, both the transmitting and receiving horns are firstly set to be polarized along *x*-axis and then the amplitude and phase of the *x*-component reflection wave could be measured. Subsequently, the polarization state of the receiving horn is changed to *y*-axis and the corresponding result of the *y*-component reflection wave could be obtained. Figure 7(b,c) shows the measured phases of the *x*- and *y*-component reflection waves at the state 1 and state 2, respectively. It is seen that the phase difference between these two component refection waves are located in range of (−90° − 20°, −90° + 20°) at the state 1 and (90° − 20°, 90° + 20°) at the state 2 in the frequency band of 3.4 GHz ~ 8.8 GHz, which agrees well with the simulation results. The characteristic of the CP waves at different states can be then calculated by using the formula of *R*_±_ = *R*_*xx*_ ± i*R*_*yx*_, where the subscript “+” indicates the RHCP wave and “−” indicates the LHCP wave. The measured LP-CP conversion performances for both two operating states are given in Fig. 7(d,e), respectively, where the simulation results are also given as comparison. It is seen that the RHCP reflection wave is produced from 3.4 GHz to 8.8 GHz at the state 1, where the isolation between RHCP and LHCP outgoing wave is larger than 15 dB (corresponding to AR ≈ 3 dB). Its reflection loss varies between 0.4 dB and 2.7 dB with an average of about 1 dB. When the meta-mirror operates at the state 2, the similar result is obtained at the same frequency band where the LHCP reflection wave is generated. The minimum reflection loss is about 0.2 dB at 6 GHz and its cross-polarization ratio is larger than 15 dB from 3.4 GHz to 8.8 GHz. There is some difference between simulated and measured results at the state 2, which is maybe due to the fabrication tolerance and measurement errors, especially for the soldering tolerance of the pin diodes. Figure 7(f) shows the measured and simulated LP reflection spectra of the sample at the state 3 where there is no bias voltage applied to this sample. It is seen that the outgoing wave can still keep the same polarization state as the incident wave. The reflection loss is less than 1.2 dB at a wide band ranging from 3.4 GHz to 8.8 GHz. Therefore, the designed meta-mirror has been experimentally verified to have three polarization states which can be dynamically controlled as required.

## Discussion

In summary, the active meta-mirror with multi-polarization function is presented. This meta-mirror integrates the PIN diodes into the design of meta-atoms. When tuning the working state of the PIN diodes, the reflection phase difference of this meta-mirror at two orthogonal directions would be dynamically switched among −90°, +90° and 0°, corresponding to three different polarization states which are LHCP, RHCP and LP states, respectively. Both simulated and measured results have verified that the designed meta-mirror has the capability of converting the incident LP wave into RHCP or LHCP reflected wave between 3.4 and 8.8 GHz where it also can keep the original LP states. The proposed active meta-mirror could be developed for several potential applications such as spin-orbit interaction^29^ and dynamic beam steering^30^.

## Additional Information

**How to cite this article**: Cui, J. *et al.* Dynamical manipulation of electromagnetic polarization using anisotropic meta-mirror. *Sci. Rep.* **6**, 30771; doi: 10.1038/srep30771 (2016).

## References

[b1] Shelby R. A., Smith D. R. & Schultz S. Experimental verification of a negative index of refraction. Science 292, 77–79 (2001). 11292865 10.1126/science.1058847

[b2] Xu T., Agrawal A., Abashin M., Chau K. J. & Lezec H. J. All-angle negative refraction and active flat lensing of ultraviolet light. Nature 497, 470–474 (2013). 23698446 10.1038/nature12158

[b3] Zhu W. M. *et al.* A flat lens with tunable phase gradient by using random access reconfigurable metamaterial. Adv. Mater. 27, 4739–4743 (2015). 26184076 10.1002/adma.201501943

[b4] Chen Y. *et al.* Engineering the phase front of light with phase-change material based planar lenses. Sci. Rep. 5, 8660 (2015). 25726864 10.1038/srep08660 PMC4345347

[b5] Luk’yanchuk B. *et al.* The Fano resonance in plasmonic nanostructures and metamaterials. Nat. Mater. 9, 707–715 (2010). 20733610 10.1038/nmat2810

[b6] Schurig D., Mock J. J., Justice B. J. & Cummer S. A. Metamaterial electromagnetic cloak at microwave frequencies. Science 314, 977–980 (2006). 17053110 10.1126/science.1133628

[b7] Cummer S. A. *et al.* Scattering theory derivation of a 3D acoustic cloaking shell. Phys. Rew. Lett. 100, 024301 (2008). 10.1103/PhysRevLett.100.024301 18232873

[b8] Liu N., Liu H., Zhu S. N. & Giessen H. Stereometamaterials. Nat. Photon. 3, 157–162 (2009).

[b9] Decker M., Zhao R., Soukoulis C. M., Linden S. & Wegener M. Twisted split-ring-resonator photonic metamaterial with huge optical activity. Opt. Lett. 35, 1593 (2010). 20479819 10.1364/OL.35.001593

[b10] Mutlu M., Akosman A. E., Serebryannikov A. E. & Ozbay E. Asymmetric chiral metamaterial circular polarizer based on four U-shaped split ring resonators. Opt. Lett. 36, 1653–1655 (2011). 21540958 10.1364/OL.36.001653

[b11] Ma X. *et al.* Dual-band asymmetry chiral metamaterial based on planar spiral structure. Appl. Phys. Lett. 101, 161901 (2012).

[b12] Mutlu M. & Ozbay E. A transparent 90° polarization rotator by combining chirality and electromagnetic wave tunneling. Appl. Phys. Lett. 100, 051909 (2012).

[b13] Ye Y. Q. & He S. L. 90 degree polarization rotator using a bilayered chiral metamaterial with giant optical activity. Appl. Phys. Lett. 96, 203501 (2010).

[b14] Huang C. *et al.* Dual-band 90 degree polarization rotator using twisted split ring resonators array. Opt. Comm. 291, 345–348 (2013).

[b15] Arnaud E. *et al.* Global design of an EBG antenna and meander-line polarizer for circular polarization. *IEEE Antennas Wireless Propag*. Lett. 9, 215–218 (2010).

[b16] Euler M., Fusco V., Cahill R. & Dickie R. 325 GHz single layer sub-millimeter wave FSS based split slot ring linear to circular polarization convertor. IEEE Trans. Antennas Propag. 568, 2457–2459 (2010).

[b17] Pu M. *et al.* Anisotropic meta-mirror for achromatic electromagnetic polarization manipulation. Appl. Phys. Lett. 102, 131906 (2013)

[b18] Guo Y. *et al.* Dispersion management of anisotropic meta-mirror for super-octave bandwidth polarization conversion. Sci. Rep. 5, 8434 (2015). 25678280 10.1038/srep08434 PMC4326699

[b19] Wei Z. Y., Cao Y., Fan Y. C., Yu X. & Li H. Q. Broadband polarization transformation via enhanced asymmetric transmission through arrays of twisted complementary split-ring resonators. Appl. Phys. Lett. 99, 221907 (2011).

[b20] Ma X. *et al.* Multi-band circular polarizer using planar spiral metamaterial structure. Opt. Express 20, 16050–16058 (2012). 22772295 10.1364/OE.20.016050

[b21] Gansel J. K. *et al.* Gold helix photonic metamaterial as broadband circular polarizer. Science 325, 1513–1515 (2009). 19696310 10.1126/science.1177031

[b22] Li X. S., Yang Y. Z., Wang J. & Zhao M. Broadband terahertz circular polarizers with single- and double-helical array metamaterials. J. Opt. Soc. Am. A. 28, 19–23 (2011). 10.1364/JOSAA.28.000019 21200407

[b23] Tao H. *et al.* Reconfigurable Terahertz metamaterials. Phys. Rev. Lett. 103, 147401 (2009). 19905602 10.1103/PhysRevLett.103.147401

[b24] Zhu W. M. *et al.* Microelectromechanical Maltese-cross metamaterial with tunable terahertz anisotropy. Nat. Commun. 3, 1274 (2012). 23232404 10.1038/ncomms2285 PMC3535344

[b25] Ma X. *et al.* An active metamaterial for polarization manipulating. Adv. Opt. Mater. 2, 945–949 (2014).

[b26] Zhang S. *et al.* Photoinduced handedness switching in terahertz chiral metamolecules. Nat. Commun. 3, 942–948 (2012). 22781755 10.1038/ncomms1908

[b27] Zhang Y., Feng Y. & Zhu B. Graphene based tunable metamaterial absorber and polarization modulation in terahertz frequency. Opt. Express 22, 22743–22752 (2014). 25321743 10.1364/OE.22.022743

[b28] Kraus J. D. & Marhefka R. J. Antennas: for all applications, Third ed., The Mc-Hill Companies, New York, NY (2002).

[b29] Pu M. *et al.* Spatially and spectrally engineered spin-orbit interaction for achromatic virtual shaping. Sci. Rep. 5, 9822 (2015). 25959663 10.1038/srep09822 PMC4426594

[b30] Luo X. Principles of electromagnetic waves in metasurfaces. Sci. China-Phys. Mech. Astron. 58, 594201 (2015).

